# Heterogeneous characteristics of γδ T cells in peripheral blood of diffuse large B-cell lymphoma

**DOI:** 10.1186/s40364-025-00795-x

**Published:** 2025-06-07

**Authors:** Peng-Lin Wang, Wen-pu Lai, Jia-Mian Zheng, Xiao-Fang Wu, Jian-Nan Zhan, Ting-Zhuang Yi, Zhen-Yi Jin, Xiu-Li Wu

**Affiliations:** 1 Institute of Hematology, School of Medicine, Jinan University, Guangzhou, China; 2 The First Affiliated Hospital of Jinan University, School of Stomatology, Clinical Research Platform for Interdiscipline of Stomatology, Jinan University, Guangzhou, China; 3 The First Affiliated Hospital, Jinan University, Guangzhou, 510632 China; 4 Key Laboratory for Regenerative Medicine of Ministry of Education, Institute of Hematology, School of Medicine, Jinan University, Guangzhou, 510632 China; 5 Department of Systems Biomedical Sciences, School of Medicine, Jinan University, Guangzhou, 510632 China; 6 Key Laboratory of Viral Pathogenesis and Infection Prevention and Control (Jinan University), Ministry of Education, Guangzhou, China; 7 Department of Oncology, Affiliated Hospital of YouJiang Medical University for Nationalities, Baise, China; 8 Department of Pathology, School of Medicine, Jinan University, Guangzhou, China; 9 Jinan-Puhua Joint Laboratory, Guangzhou, China; 10 Medical Experimental Research Center, School of Medicine, Jinan University, Guangzhou, China

**Keywords:** Diffuse large B-cell lymphoma, Single-cell RNA sequencing, Tumor immunology, Γδ T cells

## Abstract

**Background:**

Diffuse large B-cell lymphoma (DLBCL) is a highly heterogeneous disease with variable clinical and molecular features. Studies have highlighted the significant role of γδ T cells in the survival of leukemia patients. However, the heterogeneity of γδ T cells and their impact on clinical correlation in the peripheral blood of patients with DLBCL remain unclear.

**Method:**

Single-cell RNA sequencing (scRNA-seq) was employed on 9 blood samples, sourced from 6 patients with diffuse large B-cell lymphoma (DLBCL) and 3 healthy individuals (HIs), to delineate clinically pertinent γδ T cell states and subsets in DLBCL patients. Flow cytometry was then employed to validate the relationship between DLBCL prognosis and γδ T cell subsets.

**Result:**

Our study integrated genetic drivers through consensus clustering, leading to the identification of 6 distinct γδ T cell subsets in DLBCL and HIs. These subsets include a naïve γδ T cell subset characterized by TCF7 and LEF1 expression, a memory γδ T cell subset sharing common genes such as GZMK, IL7R, an anti-tumor γδ T cell subset with overexpression of IFNG, TNF, and CD69, and two subsets exhibiting TIGIT overexpression indicative of an exhausted γδ T cell phenotype. Additionally, a cytotoxic γδ T cell subset marked by increased NKG7 and GZMB levels was identified. Our results revealed that while γδ T cells possess anti-tumor capacities, their functional effectiveness is diminished due to differentiation into exhausted subpopulations. Several clusters with high cytotoxicity scores also showed elevated exhaustion scores (C13-γδ-TIGIT.1, C14-γδ-TIGIT.2), suggesting the presence of a population in DLBCL samples that is simultaneously exhausted and cytotoxic. In particular, the TIGIT.2 γδ T cell subset manifests a more pronounced exhaustion score relative to TIGIT.1 γδ T cell subset, indicating differential levels of cellular exhaustion among these groups. Our analysis reveals a significant correlation between high expression of TIGIT γδ T cell subsets and poorer patient prognoses. We also discovered unique expression profiles within these subgroups: TIGIT.1 γδ T cells are marked by elevated CXCR4 expression, contrasting with the TIGIT.2 γδ T cell subgroup which exhibits increased CX3CR1 expression. Pseudotime analysis implies a potential differentiation trajectory from naïve and GZMK γδ T cells to various terminally differentiated subsets, with genes associated with stemness (e.g., TCF-1) subsequently downregulated. These findings suggest that TIGIT.2 subset may be further along in the differentiation trajectory, potentially representing a more terminally differentiated state than TIGIT.1 subset. According to our clinical validation cohort, the TIGIT^+^ γδ T cell subset is highly expressed in patients and correlates with poor prognosis.

**Conclusion:**

We identified genetic subtypes of γδ T cells with distinct genotypic and clinical characteristics in DLBCL patients. Expression levels within these subgroups emerged as potential indicators for patient outcomes and as crucial factors in shaping therapeutic strategies. These insights significantly advance our understanding of intricate relationships among cellular subgroups and their roles in influencing disease progression and patient prognosis.

**Supplementary Information:**

The online version contains supplementary material available at 10.1186/s40364-025-00795-x.

## Introduction

Diffuse large B-cell lymphoma (DLBCL) is the most common subtype of non-Hodgkin’s lymphoma, which is a heterogeneous group of tumors that widely in biological behavior and prognosis [1]. The clinical outcome has improved with treatment protocols combining anti-CD20 monoclonal antibody rituximab with cyclophosphamide-doxorubicin-vincristine-prednisone (R-CHOP); however, relapse and drug resistance are the main challenge for current DLBCL [2]. γδ T cells possess both adaptive and innate cytotoxic effector functions, with the potential to enhance therapeutic efficacy and reduce the possibility of immune escape [3]. Consequently, γδ T cells could offer promising therapeutic prospects for DLBCL treatment.

Human γδ T cells constitute up to 5% circulating CD3^+^ T cells and exert strong non-major histocompatibility complex (MHC) restricted cytotoxicity which contributing to immunosurveillance against malignancies [4]. Upon antigen activation, both γδ T cell subtypes differentiate from naïve (Tn) to central memory (Tcm), effector memory (Tem), and terminally differentiated effector memory (Temra) cells [5, 6]. Similar to αβ T cells, effector γδ T cells can exert a directly anti-tumor effect through producing various cytokines such as perforin, granzyme B and interferon gamma (IFN-γ) [7]. Several hallmarks of γδ T cells illustrated their essential role in tumor immune surveillance and their potential interest in anti-tumor immunotherapy [8]. Clinical trials have evidenced that low numbers of γδ T cells in peripheral blood adversely affect the treatment outcomes of patients with leukemia [9]. Decreased numbers and low density of γδ T cells in hematologic malignancy patients have been linked with poor survival outcomes [9]. Yet it is worth noting that the increased frequency of peripheral blood γδ T cells have been associated with favorable prognosis in acute myeloid leukemia (AML), leading to their exploitation for cancer immunotherapy [10]. Regulatory γδ T cells or inhibitory γδ T cells can regulate the immune balance and maintain immune tolerance.

However, different γδ T cell subtypes also have been distinguished based on their varied functions and some exhibit a “pro-tumor” role harboring regulatory functions [11]. γδ T cells expressing immune checkpoint receptors (ICRs) that prevent overt activation of the immune response, a mechanism often exploited by tumor cells to shift the balance towards immunosuppression and thus evade immune response. A novel inhibitory checkpoint receptor, T cell immunoglobulin and ITIM domain (TIGIT), has received extraordinary advertence in solid and hematological tumors immunotherapy [12]. Our previous study revealed the high frequency of TIGIT^+^ γδ T cells in *de novo* AML patients and further found that higher TIGIT^+^ Foxp3^+^ γδ T cells were associated with poor overall survival [13]. Hence, reagents targeting ICRs on γδ T cells have seen recent success in developing responses to persistent antigen stimulation [14].

Currently, the single-cell RNA sequencing (scRNA-seq) technologies have been increasingly applied to characterized immune microenvironment at the single-cell resolution [15, 16]. Our previous study identified functional T cell clusters and characterized exhausted T cell populations with up-regulated gene expression of TIGIT, PD-1, LAG3, and CTLA4 [17]. However, the characterization and diverse composition of blood circulating γδ T cell remains underexplored in DLBCL, despite γδ T cells showing potential to kill cancer cells and shift the pro-tumoral tumor microenvironment into one favoring acute response and potent anti-tumoral activity. To address this issue, the characterization of single-cell transcriptomes from circulating γδ T cells is required to identify these cells exhaustively and selectively in multiple scRNA-seq datasets from DLBCL. In this study, our aim is to provide a comprehensive global perspective on the heterogeneity of γδ T cells in DLBCL, achieved through the analysis of single-cell transcriptional profiles obtained from both patients with DLBCL and healthy individuals (HIs). It is anticipated that a comprehensive investigation of the accumulated exhaustion of γδ T cells in DLBCL will contribute to the development of enhanced treatment strategies and improved outcome prediction.

## Methods

### Patients’ recruitment and sample collection

Six individuals with DLBCL (4 newly diagnosed and 2 relapsed/refractory patients) and 2 HIs were recruited at First Affiliated Hospital of Jinan University and Guangdong Provincial People’s Hospital in Guangzhou, China between 2020 and 2021. The patients were diagnosed by pathology. Peripheral blood (PB) samples were collected from each individual, immediately followed by single cell preparation as described below. The study was approved by the Ethics Committee of the Medical School of Jinan University (JNUKY-2023-0104), and with informed consent from each participant. In total, the study involved the participation of 29 DLBCL patients (13 males, 16 females; with ages ranging from 22 to 86, and a median age of 54) and 30 HIs (14 males, 16 females; with ages ranging from 17 to 80, and a median age of 51).

### Preparation of single cell suspensions

Peripheral blood mononuclear cells (PBMCs) were isolated from blood sample by Ficoll density centrifugation (Sigma Aldrich). Briefly, 10–20 mL of fresh peripheral blood was collected in EDTA anticoagulant tubes and subsequently transferred onto Ficoll. After density gradient centrifugation for 20 min at 500 × g, PBMCs settled at the plasma-Ficoll interphase were carefully collected and washed twice with PBS. PBMCs were then re-suspended with MACS buffer for subsequent sorting.

### γδ T cells isolation

The γδ T cells were sorted from PBMCs by magnetic-activated cell sorting (MACS) technology using the Anti-pan-γδ-conjugated magnetic microbeads (Miltenyi Biotec, Germany). Then, we loaded the cell suspension onto LS columns (130-050-701, Miltenyi Biotec), where the magnetically labeled γδT cells were retained. Purity was confirmed more than 50% by TCRγδ-peridinin chlorophyll A protein (PerCP-Cy5.5, Clone: B1) after collecting and washing from columns. Then, isolated γδ T cells were frozen at -80 ℃.

### Single-cell suspension Preparation

Frozen vials of isolated γδ T cells were rapidly thawed in a 37 °C water bath for 2 min, and the vials were removed when a tiny ice crystal was left. Thawed cells were mixed with 4 mL of 37 °C prewarmed 1× PBS (Thermo Fisher Scientific) supplemented with 10% FBS. Cells were centrifuged at 500× g for 10 min at room temperature. The supernatant was removed, and the cell pellet was resuspended in 3 mL 1× PBS containing 0.04% bovine serum albumin (BSA, Sangon Biotech), passed through a 40-mm cell strainer (Falcon), and then centrifuged as above. and then cells were resuspended in cell resuspension buffer at a concentration of 1,000 viable cells/mL for scRNA-seq.

### Single-cell multiplex labeling and single-cell transcriptome construction

All samples were resuspended and washed twice with PBS (with 0.1% BSA). Then, the cells were incubated in 1 mL of staining buffer (with 0.1% BSA) and counted. First, each sample was stained with Calcein AM (Thermo Fisher Scientific Cat. No. C1430) and Draq7 (Cat. No. 564904), followed by accurate determination of cell concentration and viability with the BD Rhapsody™ Scanner, and cell viability ≥ 60% were qualified. Enriched γδ T cells from each sample were sequentially labeled with the BD Human Single-Cell Multiplexing Kit (Cat. No. 633781), rinsed with BD Pharmingen™ Staining Buffer (Cat. No. 554656) to remove any excess reagents. All qualified samples were then pooled and carefully loaded into BD Rhapsody™ Cartridges, ensuring strict handling procedures were followed. The cell capture beads are then overloaded onto the cartridges in excess to maximize cell capture efficiency. Any surplus beads were thoroughly washed away to prevent interference during subsequent steps. The BD Rhapsody™ Scanner was once again utilized to detect wells containing live cells bound to capture beads, ensuring accurate identification and isolation of target cells. To prepare the sequencing libraries, whole-transcriptome amplification products were subjected to random priming PCR. This process enriched the 3′ end of transcripts linked to both the cell label and the Unique Molecular Identifier (UMI), facilitating accurate sequencing and data analysis. Quantification of the prepared sequencing libraries was carried out using a High Sensitivity DNA chip (Agilent) on a Bioanalyzer 2200. Additionally, the Qubit High Sensitivity DNA assay (Thermo Fisher Scientific) was employed to ensure precise measurement of DNA concentration. Finally, the qualified single-cell library was sequenced using the state-of-the-art Illumina Novaseq6000 system. This high-throughput sequencing platform ensured accurate and reliable sequencing data, critical for downstream bioinformatics analysis and interpretation.

### Quality control, data processing and determination of cell types

We performed multiplexing and converted the raw sequencing data into FASTQ format using the bcl2fastq tool. scRNA-seq count matrix quality control was performed to filter out low quality cells and low expression genes. Cells with less than 200 or more than 3,000 detected genes were removed. Meanwhile, cells with more than 20% of reads mapped to mitochondrial genes were removed. Moreover, only genes expressed in more than 3 cells were kept. After quality control, downstream analyses were performed using R package ‘Seurat’ [18]. scRNA-seq data were normalized using Seurat ‘NormalizedData’ function with default parameters. High variable genes were identified with parameters ‘selection.method = vst’ and ‘nfeatures = 2,000’ using ‘FindVariableFeatures’ function. Then scaled by performing ‘ScaleData’ function. ‘RunPCA’ function was performed for dimension reduction analysis, and ‘ElbowPlot’ function helped to select suitable dimensionality. Different resolution parameters for unsupervised clustering were tested to find the best numbers of clusters. Non-linear dimensional reduction was performed by ‘RunUMAP’ function. Batch effects was removed by using the ‘RunHarmony’ function of R package ‘harmony’ [19]. before clustering analysis in Seurat. In total, 23,533 PBMCs were annotated as different major cell types based on their average gene expression of well-known marker genes, including T and NK cell (*CD3D*, *NKG7*), B cell (*CD19*, *CD79A*) and myeloid cell (*LYZ*, *CST3*). Next, T and NK cell cluster was subset using Seurat ‘subset’ function. After that, 17,571 T and NK cells were acquired and analyzed using ‘Seurat’ and ‘harmony’ packages as above. According to expression of marker genes, T and NK cells were grouped into 16 cell types. In addition, we collected published scRNA-seq data of healthy human peripheral γδ T cells and re-annotated the cells based on the expression of marker genes according to the clustering described in the original publication [20].

### Calculation of functional gene module score

To evaluate the potential functions of interest for cell clusters, the enrichment scores of functional gene modules were calculated by using ‘AddModuleScore’ function in ‘Seurat’ at single cell level. The average expression levels of the corresponding cluster or group were subtracted by the aggregated expression of control gene sets. The functional modules included genes for inferring cell cytotoxicity (*KLRF1*, *GNLY*, *CTSW*, *NKG7*, *KLRD1*, *GZMA*, *ADGRG1*, *CST7*, *KLRK1*, *FASLG*, *HCST*, *KLRB1*, *ITGB1*, *GZMB*, *PRF1*) and exhaustion (*HAVCR2*, *LAG3*, *TIGIT*, *CTLA4*, *PDCD1*, *LAYN*, *BTLA*, *CD16*, *TOX*, *HLA-DRA*, *HLA-DRB1*, *CXCL13*) scores.

### Pathway enrichment analysis

Gene Ontology (GO) and Kyoto Encyclopedia of Genes and Genomes (KEGG) enrichment analysis was conducted using R package ‘clusterProfiler’ [21]. ‘FindMarkers’ function of Seurat with parameters ‘min.pct = 0.1’ and ‘logfc.threshold = 0.25’ was used to identify DEGs. Then, ‘enrichGO’ and ‘enrichKEGG’ functions were used for pathway enrichment analysis of these genes. Gene symbols was converted using the ‘bitr’ function before pathway enrichment if necessary.

### GSVA analysis

Pathway analyses were predominantly performed on the GO and KEGG pathways described in the molecular signature database (MSigDB) [22]. Pathway activity estimates were obtained using the GSVA [23]. The GSVA algorithm obtains the pathway enrichment score matrix based on the given gene expression matrix and the marker gene set downloaded from MSigDB. Then we used the lmFit analysis of the ‘limma’ [24]. package to obtain the differential pathways. *p* value was calculated by limma and FDR was also inferred.

### SCENIC analysis

To assess transcription factor regulation activity, we applied the Single-cell regulatory network inference and clustering (SCENIC) workflow [25]. The SCENIC analysis included four steps: [1] the single-cell gene expression count table for cells from each subtype of γδ T cells was first fed into SCENIC with a list of 1,390 known human TFs (https://scenic.aertslab.org/), and sets of genes that are co-expressed (either positively or negatively) with TFs were identified by random forest models; [2] putative target genes in each coexpressed module were then subjected to cis-regulatory motif discovery analysis, and only modules with significant motif enrichment in the ± 500-bp or ± 10-kb region around the transcription start site (TSS) for the corresponding TF were kept for further analysis; [3] the AUCell algorithm of SCENIC was used to score the activity of each regulon in each cell, and regulons with average AUC score (that is, regulon activities) ≥ 0.05 were retained; and [4] regulon specificity score (RSS) of γδ T cell subtype was calculated by ‘regulon_specificity_scores’ function.

### Reconstructing cell development trajectories

To explore the developmental progression of the γδ T cell subset, R package ‘Monocle’ [26]. was used for reconstructing their development trajectories. In detail, the raw counts for cells in each cell types were extracted and normalized by the ‘estimateSizeFactors’ and ‘estimateDispersions’ functions with the default parameters. Then, ‘differentialGeneTest’ function was used to select top 1,900 significant genes (ordered by Q value) of γδ T cell for cell fate trajectory reconstruction. Cell fate trajectory of γδ T cell was split into three different cell fates. Top 100 genes that had most significantly correlated (or anti-correlated) expression profile (ordered by Q value) to each cell fate were selected using ‘differentialGeneTest’ function. Differential genes between the three branch states were placed into three groups by expression pattern using the ward.D2 clustering algorithm. Meanwhile, GO enrichment analyses were performed on genes in different clusters. Finally, the expression heatmap of top 100 genes correlated (or anti-correlated) to the γδ T cell fate pseudotime was visualized using ‘plot_pseudotime_heatmap’ function.

### Flow cytometry

PBMC, derived from blood samples of DLBCL patients and HIs, were stained for flow cytometric analysis using 3-color staining combinations with the monoclonal antibodies CD3-allophycocyanin (APC-Cy7) (Biolegend, Clone: UCHT1, 300317), TCRγδ-peridinin chlorophyll A protein (PerCP-Cy5.5) (Biolegend, Clone: B1, 331223), TIGIT-phycoerythrin (PE) (Biolegend, Clone: A15153G, 372704). Whole blood samples were incubated with an excess amount of monoclonal antibody at 4 °C for 20 min in the darkness. After the red blood cell lysis, the solution was centrifuged by 447×g for 5 min and the supernatant was discarded, followed by a PBS wash (centrifuged by 447×g, 5 min). The wash step was repeated once and single-cell suspensions were resuspended in 250 µL PBS. Data were acquired with a FACS Verse flow cytometer (BD, USA) and analyzed with FlowJo software. The percentages of γδ T cells were initially obtained by gating on the CD3 T-cell population. Subsequently, a gate was applied to the γδ T-cell subtype to determine the expression of TIGIT on the T-cell subtypes. In the course of determining gates, appropriate negative controls (unstained cells and isotype-matched controls, Biolegend, San Diego, USA) and positive controls (single stained antibody) were used.

### Validation of biomarker expression by quantitative Real-time polymerase chain reaction

Total RNA was extracted from the PBMCs using TRIzol reagent (Invitrogen, Carlsbad, CA, USA) according to the manufacturer’s instructions and reverse transcribed into complementary DNA (cDNA) using the PrimeScript™ RT reagent Kit (Takara, Japan) according to the experimental instructions. The relative expression levels of *CCL3*, *CCL4*, *CCL5*, and *CCR5* were measured by quantitative real-time polymerase chain reaction with SYBR Master Mix (TIANGEN, Beijing, China), and *B2M* was selected as an internal control. The expression levels of *CD69*, *CX3CR1*, *CXCR4*, and *GZMB*, *IFNG*, *PRF1* and *TNF* are presented as 2^−ΔCT^. The primer sequences in this study are listed as follows:

**Table:** 

CD69-F	TGCCATCAGACAGCCATGTT
CD69-R	ACCCTGTAACGTTGAACCAGT
GZMB-F	TCAAAGAACAGGAGCCGACC
GZMB-R	CGCACTTTCGATCTTCCTGC
TIGIT-F	TGGTGGTCATCTGCACAGCAGT
TIGIT-R	TTTCTCCTGAGGTCACCTTCCAC
IFNG-F	GAGTGTGGAGACCATCAAGGA
IFNG-R	GGACATTCAAGTCAGTTACCGAA
PRF1-F	CGAGTGGCTCTTCTCAGCAA
PRF1-R	GCTGCGAAATTCACTCCCAG
TNF-F	GCCCATGTTGTAGCAAACCC
TNF-R	GGAGGTTGACCTTGGTCTGG

### Statistical analysis

All statistical analyses were performed in R (version 4.1.0) and SPSS (version 13.0). *P* value less than 0.05 was considered statistically significant. According to data distribution, we used the Wilcoxon rank sum test as appropriate based on distributional assumptions.

## Results

### Identification of the γδ T cell subtypes of DLBCL by scRNA-seq

**Fig. 1:**
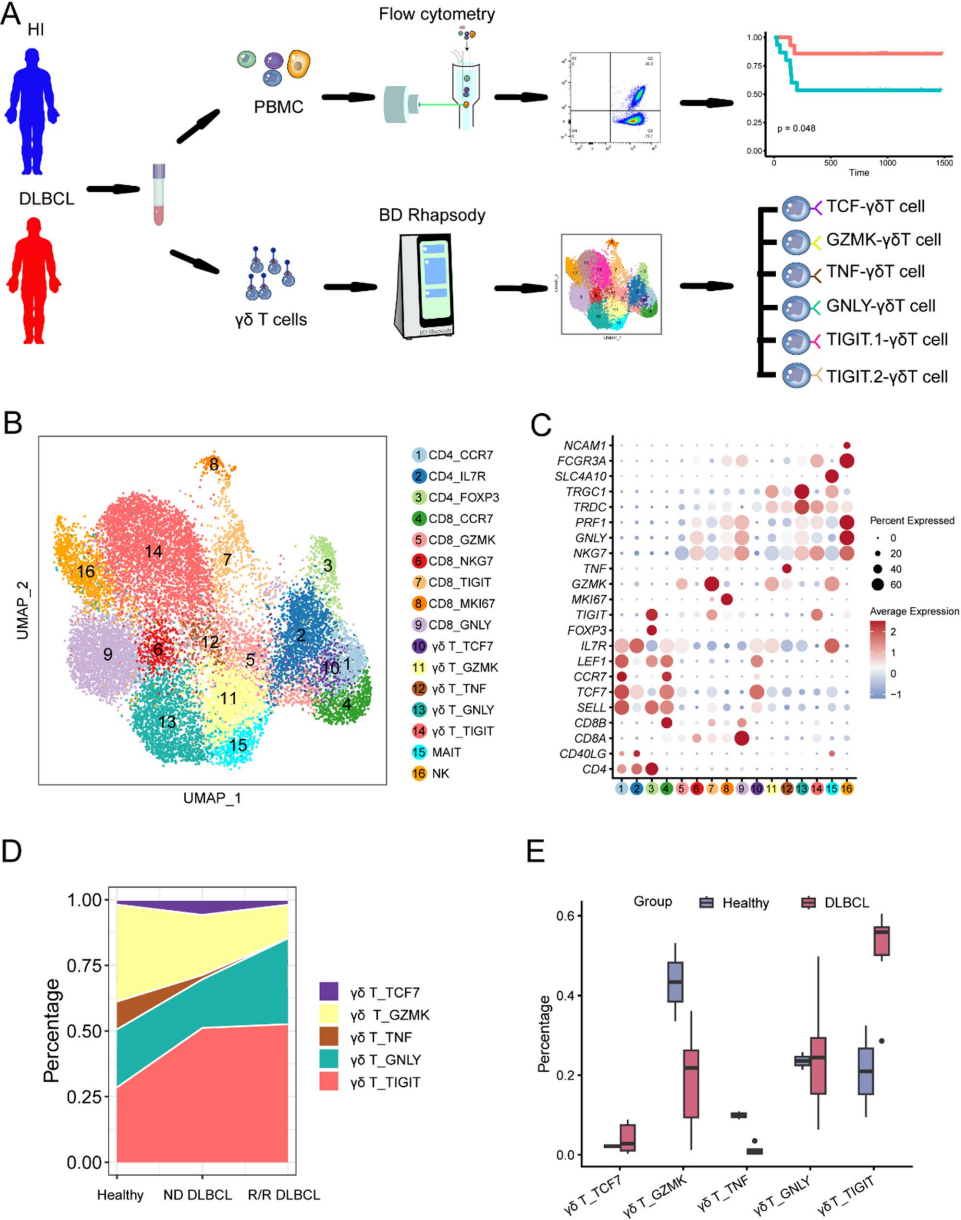
Identification of γδ T cells of patients with DLBCL and HIs through scRNA-seq. (**A**) Schematic of the overall study design. (**B**) UMAP visualization of T and NK cells from peripheral blood analyzed by scRNA-seq showing 17 major subtypes. Clusters are colored and labeled according to different cell subtypes. (**C**) The dot plot of marker genes for each cell cluster. Color-scale represents the mean normalized expression of marker genes within each cell type, and the size of the dots corresponds to the percentage of cells expressing the marker genes within each cell cluster. (**D**) Fluctuation in the percentage of each γδ T subtype across HI, ND DLBCL and R/R DLBCL groups. (**E**) Boxplots comparison of γδ T subset proportions between DLBCL patients and HIs

To illuminate the complexity of γδ T cell subtypes within DLBCL, we employed scRNA-seq to probe a cohort of 6 DLBCL patients and 2 HIs (Fig. 1A). Additionally, we performed unsupervised clustering analysis to define groups of cells with similar expression profiles. Each cluster was identified as a specified cell subpopulation according to the expression of the most variable genes and the canonical markers, including T/NK cell (gene markers: *CD3D*, *NKG7*), B cell (*CD19*, *CD79A*) and myeloid cell (*LYZ*, *CST3*) (Fig. S1E). In order to identify γδ T cells from T/NK cell cluster, we grouped all T and NK cells into 16 distinct subtypes with clustering analysis. These encompassed: CD4^+^ T cells (*CD3*^+^*CD8A*^−^*CD4*^+^, clusters 1, 2, 3); CD8^+^ T cells (*CD3*^+^*CD8A*^+^*CD4*^−^, clusters 4, 5, 6, 7, 8, 9); γδ T cells, defined by a requisite co-expression of a positive gene set (*CD3D*, *CD3E*, *TRDC*, *TRGC1* and *TRGC2*) and the exclusion of a negative gene set (*CD8A* and *CD8B*) (clusters 10, 11, 12, 13, 14); mucosal-associated invariant T cells (MAIT) characterized by *SLC4A10* (cluster 15), and NK cells (*CD3D*^*−*^, *CD3E*^−^, *NCR*^*+*^, *NCAM1*^*+*^, *NKG7*^+^, *GNLY*^+^) (cluster 16) (Fig. 1B and C).

Furthermore, given the potential functional heterogeneity of γδ T cells, a total of 5 distinct clusters representing 5 different functional γδ T cell subtypes are identified. The C10-γδ-TCF7 subtype was characterized by the high expression level of *TCF7* and *LEF1* commonly identified as naïve γδ T cells. The C11-γδ-GZMK, defined by high expression of *GZMK* and *IL7R*, thus likely representing memory γδ T cells. Additionally, the C12-γδ-TNF subtype specifically enriched for *TNF*, promoting the activation of naïve and effector T cells, is mainly exerting activation function. The C13-γδ-GNLY subtype exhibited the expression of cytotoxic molecule *NKG7* and *GNLY* which indicated the status of cytotoxic γδ T cells. Furthermore, the C14-γδ-TIGIT subtype, expressed high levels of exhaustion marker *TIGIT*, suggestive of the identity of exhausted γδ T cells (Fig. 1B and C). An interesting yet converse observation was made regarding patients with DLBCL, especially those with relapsed or refractory (R/R) DLBCL. Not only did they present an increase in circulating C14-γδ-TIGIT, but they also exhibited a reduction in circulating C11-γδ-GZMK and C12-γδ-TNF (Fig. 1D and E). Moreover, we analyzed a large-scale scRNA-seq dataset of peripheral γδ T cells from healthy individuals and identified a similar TIGIT^+^ γδ T cell subset (cluster2 and 3, Fig. S2A-B) [20]. Notably, the proportion of TIGIT^+^ γδ T cell was consistent with our results, showing a lower frequency in healthy individuals compared to DLBCL patients (Fig. S2C). The aforementioned results imply more than a possibility that DLBCL may tend to favor an enrichment in C14-γδ-TIGIT, along with a decrease in C12-γδ-TNF and C11-γδ-GZMK; they imply a potential association between C14-γδ-TIGIT and poor prognosis specifically.

### Assessing functional heterogeneity through calculation of cytotoxic and exhausted immune scores for γδ T cells

**Fig. 2:**
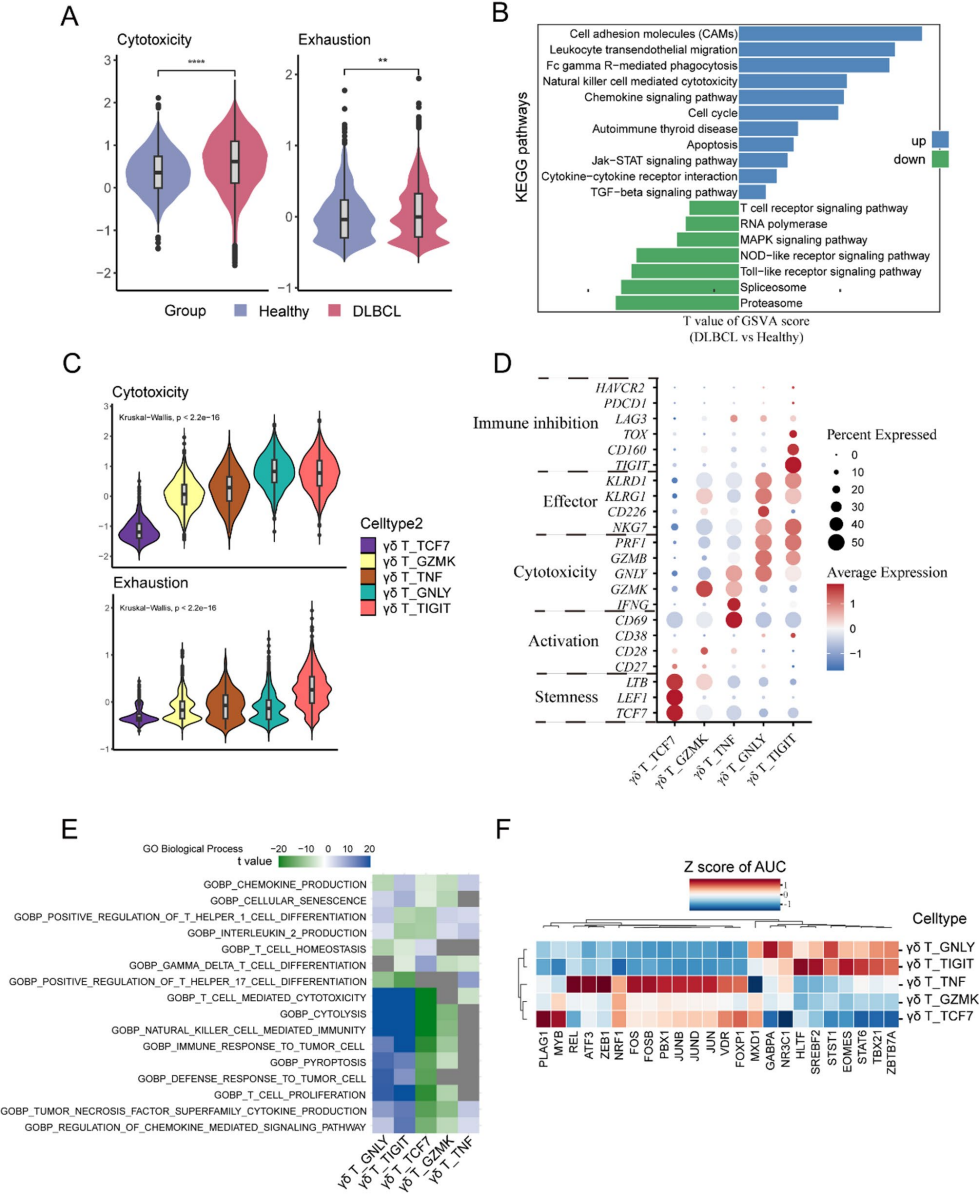
The functional characteristics and expression differences of specific genes across various γδ T-cell subtypes. (**A**) The functional characteristics and expression differences of specific genes across various γδ T-cell subtypes. Violin plots with boxplot insert showed the comparison of the cytotoxicity (left panel) and exhausted (right panel) scores of γδ T cells between DLBCL patients and HIs. **P < 0.01, ****P < 0.0001; *P*-values were calculated by Wilcoxon Rank Sum test (two-sided). (**B**) Comparation of KEGG signaling pathway activities scored per cell by GSVA for γδ T cells between sample group (DLBCL patients vs. HI). Bar plots represented the up-regulated (blue) and down-regulated (green) pathways for significantly enriched pathways in DLBCL patients. (**C**) Comparison of the cytotoxicity (upper panel) and exhausted (lower panel) scores among each γδ T cell subtype. **P < 0.01; ****P < 0.0001. *P*-values were calculated by Kruskal-Wallis test. (**D**) The dot plot of functional genes for each cell cluster. Color-scale represents the mean normalized expression of functional genes within each cell type, and the size of the dots corresponds to the percentage of cells expressing the functional genes within each cell cluster. (**E**) Gene ontology (GO) biological process activities scored per cell by GSVA for γδ T cells between sample group (DLBCL patients vs. HI). Heatmap modules exhibited up-regulated (indicated by the color blue) or down-regulated (indicated by the color green) for significantly enriched pathways in each γδ T cell subtypes. (**F**) The heatmap reflects cell-type-specific TF regulon activities at single-cell resolution, (blue represents high-regulon activity, red represents low-regulon activity)

To evaluate the functional heterogeneity of γδ T cells, the cytotoxic and exhausted immune scores for γδ T cells were calculated. The γδ T cells in DLBCL patients exhibited a higher cytotoxic and exhausted score (*P* < 0.05) when compared with HIs (Fig. 2A). Furthermore, we performed GSVA analysis of KEGG signaling pathway to gain more insights into the functional divergence of γδ T cells: The status of DLBCL disease upregulates a list of genes in γδ T cells categorized into signaling pathways, including cell apoptosis, TGF-β signaling pathway (Fig. 2B). Conversely, the pathways associated with cell activation are downregulated (e.g., T cell receptor signaling pathway) (Fig. 2B). In general, these results confirm that γδ T cells possess a more potent immunosuppressive capacity in DLBCL patients compared to HIs; however, given the tumor-specific differences in γδ T cell subtypes, it remains to be investigated whether there was significant functional heterogeneity among γδ T cell subsets, particularly those accounting for the high levels in patients.

Thus, the cytotoxicity and exhausted immune scores for each γδ T cell subset were calculated, respectively. Compared with other subsets, C13-γδ-GNLY and C14-γδ-TIGIT possessed a higher cytotoxicity score, while C14-γδ-TIGIT also demonstrated a higher cell exhaustion score (*P* < 0.001) (Fig. 2C). As depicted in dot plot of typical functional genes among each cluster, C10-γδ-TCF7 expressed high level of naive genes (*LTB*, *LEF1*, *TCF7*, *SELL*, *CCR7*), C11-γδ-GZMK expressed high level of GZMK and C12-γδ-TNF expressed high level of *GZMK*, *IFNG*, *CD69* (Fig. 2D). Moreover, C14-γδ-TIGIT and C13-γδ-GNLY both exhibited high expression for various genes associated with effector function (*KLRD1*, *KLRG1*, *NKG7*), cytotoxicity (*PRF1*, *GZMB*), yet it is noteworthy to mention that the genes associated with immune inhibition (*TOX*, *CD160*, *TIGIT*) are higher expressed in C14-γδ-TIGIT (Fig. 2D). Our preliminary qPCR validation confirmed a lower expression of effector molecules, including perforin, IFNG, TNF, and the activation marker CD69 in patients (Fig. S5C). To further probe the functional heterogeneity of γδ T cells, GO enrichment analysis was performed. C14-γδ-TIGIT and C13-γδ-GNLY are shared with some enriched functions related to T cell mediated cytotoxicity, natural killer cell mediated immunity, pyroptosis, and cytolysis (Fig. 2E). Whereas, compared to C14-γδ-TIGIT, C13-γδ-GNLY additionally involved in gamma delta T cell differentiation (Fig. 2E). According to regulatory program analysis, the regulon activities of prioritized TFs are similar in C14-γδ-TIGIT and C13-γδ-GNLY (including HLTF, SREBF2, STSY1, EOMES, STAT6, TBX21, ZBTB7A). The regulon activities of FOS, FOSB, PBX1, JUNB, etc. are significantly higher in C12-γδ-TNF, and the regulon activities of PLAG1, MYB are significantly higher in C10-γδ-TCF7 (Fig. 2F). Interestingly, not only did C14-γδ-TIGIT exhibit immune inhibition but also cytotoxicity, indicating a complex cell state. In light of these results, we uncovered a more cytotoxic and exhausted function among patients’ γδ T cells. This may be attributed to their high proportion of C14-γδ-TIGIT, which were more exhausted than other γδ T cell subtypes yet exhibited cytotoxicity comparable to that of C13-γδ-GNLY, arising from the high expression of cytotoxic, effector and immune inhibition genes.

### Functional characterization of the TIGIT.1 and TIGIT.2 γδ T cells in DLBCL

**Fig. 3:**
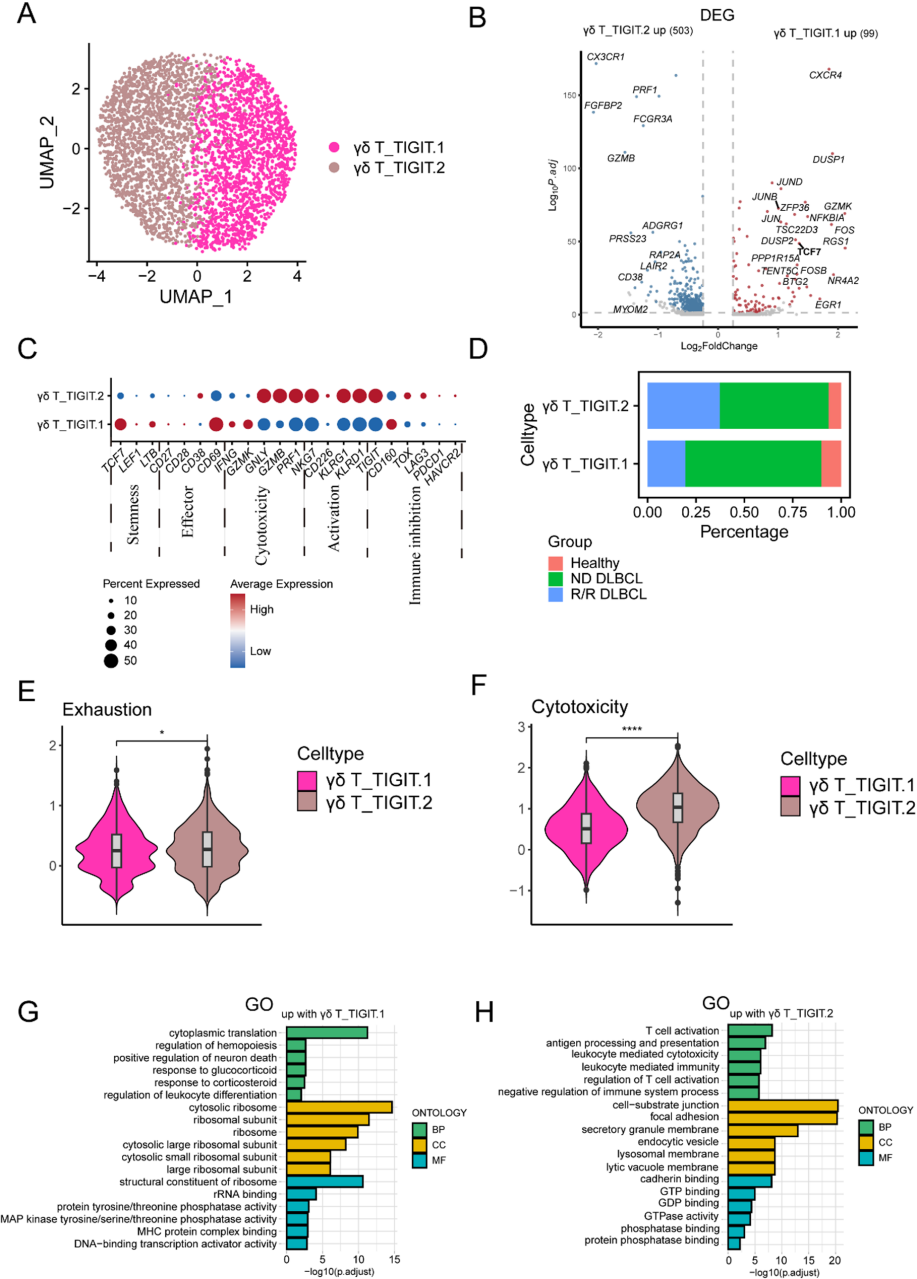
The functional characteristics between TIGIT.1 and TIGIT.2 γδ T-cell subtypes. (**A**) UMAP visualization TIGIT.1 γδ T cell subtype and TIGIT.2 γδ T cell subtype after reclustering of C14-γδ-TIGIT from DLBCL patients and HIs. (**B**) Volcano plots showing significantly differentially expressed genes (DEGs) (Fold change > 1.5, two-part hurdle model, adjusted *p*-value < 0.05, Bonferroni correction) in TIGIT.1 vs. TIGIT.2 γδ T cells. (**C**) The dot plot of functional genes for TIGIT.1 and TIGIT.2 γδ T cells. Color-scale represents the mean normalized expression of functional genes within each cell type, and the size of the dots corresponds to the percentage of cells expressing the functional genes within each cell cluster. (**D**) Variation of the proportion of TIGIT.1 and TIGIT.2 γδ T cells among HI, ND DLBCL and R/R DLBCL groups. (**E**) Violin plots with boxplot insert showed the comparison of the cytotoxicity (right panel) and exhausted (left panel) scores between TIGIT.1 and TIGIT.2 γδ T cells. *P < 0.05, ****P < 0.0001; *P*-values were calculated by Wilcoxon Rank Sum test (two-sided). (**F**) Violin plots with boxplot insert showed the comparison of the cytotoxicity (right panel) and exhausted (left panel) scores between TIGIT.1 and TIGIT.2 γδ T cells. *P < 0.05, ****P < 0.0001; *P*-values were calculated by Wilcoxon Rank Sum test (two-sided). (**G**) Gene Ontology (GO) term enrichment analysis. Significantly enriched GO terms were selected based on FDR < 0.05. GO terms of the categories of Biological Processes, Cellular Components, and Molecular Functions are depicted in green, yellow, and blue, respectively

We then focused on further characterization of C14-γδ-TIGIT and reclustered them into two subtypes, γδ_TIGIT.1 (CXCR4 high expression) and γδ_TIGIT.2 (CX3CR1 high expression) (Fig. 3A and B). Along the analysis of DEGs, accounting for some functional specific genes, γδ_TIGIT.1 highly expressed *TCF7*, *LTB*, *CD69*, *IFNG*, *GZMK*, *CD160*, while γδ_TIGIT.2 exhibited high expressions in *GNLY*, *GZMB*, *PRF1*, *NKG7*, *KLRG1*, *KLRD1*, *TIGIT*, *TOX*, *PDCD1* and *LAG3* (Fig. 3C). There was a higher percentage of γδ_TIGIT.1 in the HIs and ND DLBCL patients whereas γδ_TIGIT.2 constituted the majority in R/R DLBCL patients (Fig. 3D). The increased cytotoxicity and exhaustion scores in γδ_TIGIT.2 potentially imply that this subtype is cytotoxic and exhausted at the same time (Fig. 3E and F). Furthermore, the RSS presented the top novel candidate TFs of γδ_TIGIT.1 (top 6: *FOSB*, *NRF1*, *JUN*, *ATF3*, *FOS*, and *JUNB*) (Fig. S4A) and γδ_TIGIT.2 (top 6: *TBX21*, *STAT1*, *EOMES*, *ZBTB7A*, *SREBF2*, and *POLR2A*) (Fig. S4B). The analysis of co-expression network identified the novel candidate TFs (ATF3, BCLAF1, BHLHE40, CEBPB, CFL2, CHD2, EGR1, ELF1, ELF2, ELF4, ELK4, EOMES, ETS1, FOS, FOSB, IRF2, JUN, JUNB, JUND, KDM5A, KLF2, KLF6, KLF9, MXI1, NFIL3, POLR2A, RELA, RUNX3, SRF, YY1) that co-expressed to regulate the marker gene co-expression (notably, *CXCR4* and *TIGIT*) in γδ_TIGIT.1 (Fig. S4C). The analysis of co-expression network identified the novel candidate TFs (*POLR2A*, *KLF2*, *SRF*, *RUNX3*, *IRF2*, *YY1*, *KDM5A*, *ETS1*, *ELF4*, *ELK4*, *RELA*) that concomitantly regulate the marker gene co-expression (notably, *CX3CR1* and *TIGIT*) in γδ_TIGIT.2 (Fig. S4D). The GO enrichment results revealed that the enriched functions and signaling pathways of γδ_TIGIT.1 were related to differentiation, such as “cytosolic ribosome”, “regulation of leukocyte differentiation”. Furthermore, γδ_TIGIT.2 were of genes upregulated in “negative regulation of immune system process”, “T cell activation”, “leukocyte mediated immunity” (Fig. 3G and H). In comparison, γδ_TIGIT.1 might still potentially be endowed with cell stemness, while γδ_TIGIT.2 might play a dichotomous role in terms of cytotoxic mediator (*GZMB*, and *GNLY*) expression and IFN-γ production.

### Potential transition from Naive γδ T cells to terminally differentiated γδ T cells revealed by scRNA-Seq

**Fig. 4:**
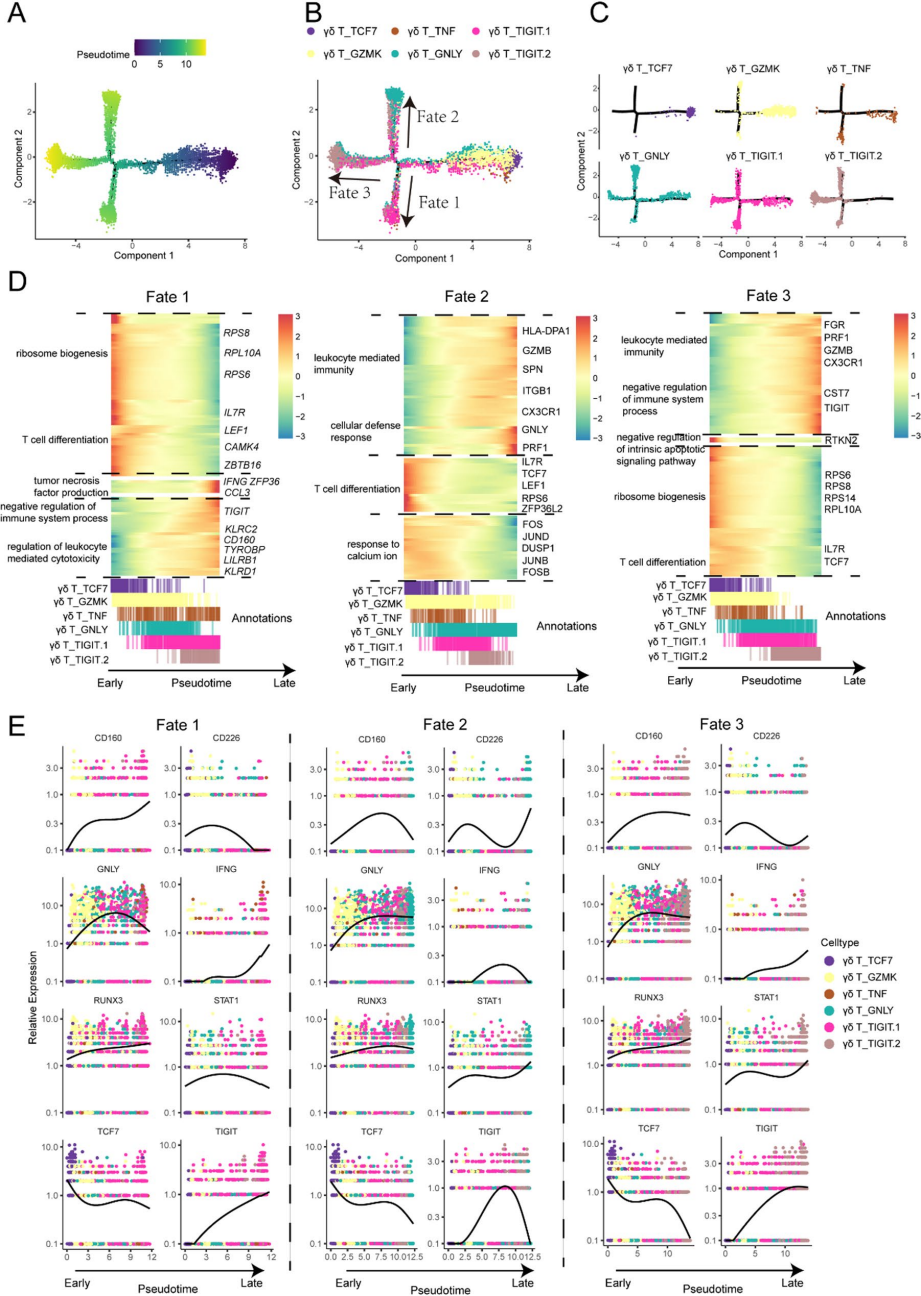
Cell fate trajectory of γδ T cells. (**A**) DDR (discriminative dimensionality reduction) tree visualization of γδ T subtype trajectory. (**B**) DDR (discriminative dimensionality reduction) tree visualization of γδ T subtype trajectory with cell type. (**C**) The distribution of each γδ T cell subsets in DLBCL patients during development of DDR trees. (**D**) Heat map displaying the differentially expressed genes across γδ T cell pseudotime trajectory. The x-axis represents pseudo-temporal ordering, with gene expression levels normalized to their maximum values and smoothed across the pseudotime axis. Genes are categorized based on their functions and expression patterns. The bottom portion of the heat map includes cell type annotations for cells aligned along the pseudotime axis. (**E**) The dynamic genes during the cell differentiation were visualized for fate1 (middle), fate2 (left panel), and fate3 (right panel). Each dot indicates a single cell colored by its cluster, the solid lines showed smoothed expression curves of representative genes along the trajectory

To further investigate the differentiation direction of γδ T cells in DLBCL, we identified potential relationships among each state by cell fate trajectory analysis. The ordering of cell fate trajectories for all γδ T cells yields a combination of 6 states, branching into 3 primary cell fates (Fig. 4A and B). Mapping of the γδ T cells along the trajectory, it became apparent that the C10-γδ-TCF and C11-γδ-GZMK primarily occupied the tip of the initial trajectory. In contrast, the C12-γδ-TNF and primarily aligned with the branches corresponding to cell fate 1 (Fig. 4B and C). Cell fate 2 was significantly enriched with C13-γδ-GNLY while γδ_TIGIT.2 cells are primarily localized toward the end of the trajectory of fate 3 (Fig. S4A and B). γδ_TIGIT.1 cells are located between the main trunk and fate 3, suggesting that they may represent a transitional state between the main lineage and exhaustion, potentially corresponding to pre-exhausted progenitor cells. Apparently, the majority of their locations are situated in close proximity to the terminal stages of differentiation.

Of particular note, to offer a nuanced visualization of how gene expression patterns evolve with the γδ T cell developmental trajectory, we selected the top 100 genes that had most significantly correlated (or anti-correlated) expression profile (ordered by Q value) to each cell fate (Fig. 4D). Along the progression of the pseudo-time axis (specifically, fate 1), we observed a decline in the expression of genes related to ribosome biogenesis (e.g., *RPS8*, *RPL10A*, *RPS6*), T cell differentiation (e.g., *IL7R*, *LEF1*, *CAMK4*, *ZBTB16*). Conversely, genes associated with tumor necrosis factor production (e.g., *IFNG*, *ZFP36*, *CCL3*), negative regulation of immune system process (e.g., *TIGIT*) and regulation of leukocyte mediated cytotoxicity (e.g., *KLRC2*, *CD160*, *TYROBP*, *LILRB1*, *KLRD1*) manifested a progressive upswing along the trajectory of fate 1 (Fig. 4D). Along the pseudotime trajectory fate 2, we observed an increase of genes related to leukocyte mediated immunity (e.g., *HLA-DPA1*, *GZMB*, *SPN*, *ITGB1*) and cellular defense response (e.g., *CX3CR1*, *GNLY*, *PRF1*), while a list of genes correlated with T cell differentiation (e.g., *IL7R*, *TCF7*, *LEF1*, *RPS6*, *ZFP36L2*) and response to calcium ion (e.g., *FOS*, *JUND*, *DUSP1*, *JUNB*, *FOSB*) were decreased. As C10-γδ-TCF and C11-γδ-GZMK differentiated towards γδ_TIGIT.2 (cell fate 3), genes involved in leukocyte mediated immunity (e.g., *FGR*, *PRF1*, *GZMB*, *CX3CR1*) and negative regulation of immune system process (e.g., *CFT7*, *TIGIT*) are increased. Additionally, the genes implicated in negative regulation of intrinsic apoptotic signaling pathway (e.g., *RTKN2*), ribosome biogenesis (e.g., *RPS6*, *RPS8*, *RPS14*, *RPL10A*) and T cell differentiation (e.g., *IL7R*, *TCF7*) (Fig. 4D).

The smoothed expression curves for functional genes alone each cell trajectory was of special interest. Immune inhibition-related genes, such as *CD160* and *TIGIT* were highly expressed along cell fate 1 and 3, while activation-related gene CD226 was up-regulated along cell fate 2 and 3. Cytotoxic-related gene *GNLY* was declined substantially along cell fate 1 and 3, but notably so in cell fate (1) However, another effector gene, *IFNG*, was increased along cell fate 1 and 3, as opposed to cell fate (2) Cell stemness-related gene *TCF7* was decreased along all cell fates, indicating a differentiation towards terminal cell states (Fig. 4E).

With the temporal dynamics of γδ T cell states across differentiation, it can be inferred that C10-γδ-TCF and C11-γδ-GZMK may serve as progenitor γδ T cells and may potentially leading to three directions of terminal differentiation, enriched with C13-γδ-GNLY, γδ_TIGIT.2 and C12-γδ-TNF. Since Fate 1 possessed γδ_TIGIT.1 at the end of the branch, there may be a transition potential between naïve and exhaustion.

### γδ T cell subtype distribution in peripheral blood from DLBCL patients and his

**Fig. 5:**
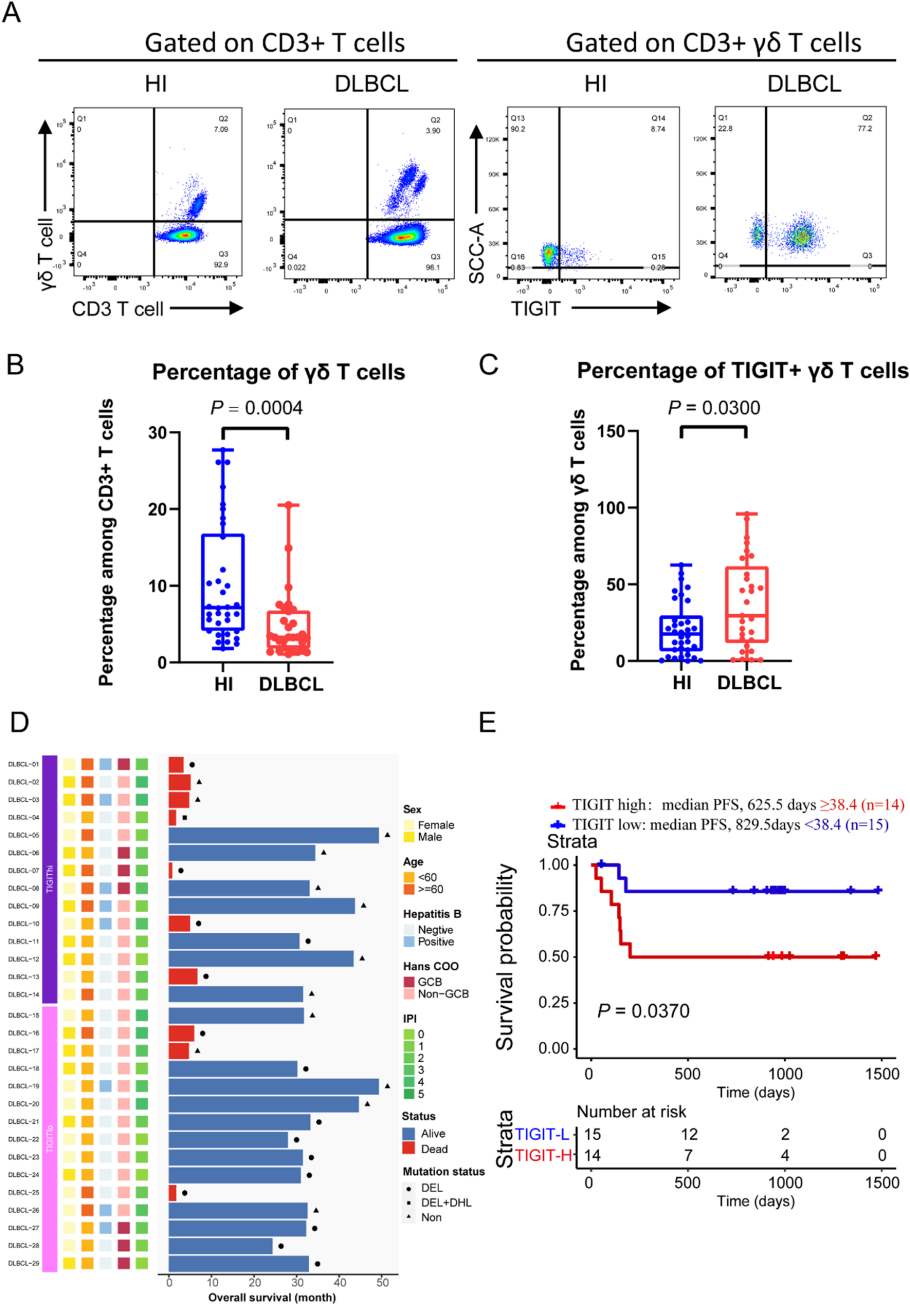
TIGIT expression levels on γδ T cells in determining the prognosis of patients with DLBCL. (**A**) Gating strategy for γδ T cells sorting in peripheral blood. (**B**) Percentage of γδ T cells in the peripheral blood of HIs and DLBCL patients. (**C**) Percentage of TIGIT^+^ γδ T cells in the peripheral blood of HIs and DLBCL patients. (**D**) The swimmer plot visually represents the impact of both clinical and molecular characteristics on patient survival. Horizontal bars indicate the duration of survival in months, while data points signify the outcomes for individual patients. The data matrix on the left matches patient identifiers with their corresponding clinical and molecular attributes. On the right side of the diagram, a concise visual summary of survival outcomes correlated with each factor is provided. This offers vital insights into the prognostic significance of each variable under consideration. (**E**) The PFS (progress free survival) of DLBCL patients; the optimal cutoff for PFS in relation to the expression of TIGIT was determined to be 68.6%

To validate the findings from scRNA-seq analysis, flow cytometry was performed on PBMCs derived from the blood samples of DLBCL patients and HIs (Fig. 5A). A comprehensive synthesis pertaining to the proportion of γδ T cells within CD3^+^ T cell population, along with the expression of TIGIT in γδ T cells, is concisely presented (Fig. 5B and C). The proportion of γδ T cells significantly decreased in DLBCL, while the proportion of TIGIT^+^ γδ T cells significantly increased (*P* = 0.0004, 0.03). The clinical data are summarized (Fig. 5D). To confirm whether the TIGIT expression is associated with cancer progression and/or outcomes, samples were segregated into 2 groups based on TIGIT median expression: high expression (*n* = 14); and low expression (*n* = 15) (Fig. 5D). The Kaplan-Meier survival analysis demonstrated that patients with higher TIGIT expression experienced the poorer outcomes, as evidenced by a reduced time to progression-free survival (PFS) (*P* = 0.037) (Fig. 5E). The average proportion of PD-1^+^ γδ T cells were not statistically significantly different between γδ_TIGIT.1 (TIGIT^+^ CXCR4^+^ γδ T cells) and γδ_TIGIT.2 (TIGIT^+^ CXCR4^−^ γδ T cells) (Fig. S5A). However, the proportion of PD-1^+^ γδ_TIGIT.1 and γδ_TIGIT.2 cells were lower in HIs than in DLBCL patients (Fig. S5B and C). Furthermore, we performed preliminary validation of key genes and proteins through quantitative PCR. Our data claimed that the PBMC from DLBCL possess a lower expression of effector molecules such as *perforin*, *IFNG*, *TNF* and activated marker *CD69* (Fig. S5D). Moreover, the expression of *CXCR4* in DLBCL were higher while *CX3CR1* were lower.

## Discussion

In this study, we utilized single cell RNA-sequencing technique to systematically explore the complex immune landscape of DLBCL focusing on γδ T cells. This method facilitated: [1] revealing 6 subtypes of γδ T cells (C10-γδ-TCF, C11-γδ-GZMK, C12-γδ-TNF, C13-γδ-GNLY and C14-γδ-TIGIT.1 and C14-γδ-TIGIT.2) [2], observing the heterogeneity in gene expression among γδ T cell subtypes [3], hypothesizing different roles of each γδ T cell subtype (stemness [27], memory T cell function [28], activation [29], cytotoxicity [30, 31], immunosuppression [32, 33], etc.) [4] predicting the differentiation trajectory of γδ T cells, and exploring a potential relationship between TIGIT^+^ γδ T cells and negative outcomes in DLBCL patients.

γδ T cells represent an evolutionarily conserved group of innate lymphocytes characterized by significant functional heterogeneity, performing both active immune responses and immunosuppression across tumor progression [34]. It is commonly known that the upregulation of tumor necrosis factor (TNF) subsequent to immune-cell activation indicates an active involvement in modulating immune responses [35]. while tumor immune evasion is attributed to the loss of TNF sensitivity [36]. Our results are in agreement with this, as circulating C12-γδ-TNF was observed to trend toward decline in DLBCL compared with HIs.

Additionally, among the previous study, the role of TIGIT in pancreatic cancer and hematological malignancies has been related to anti-inflammatory and exhausted phenotypes as a consequence of tumor progression and negative patient outcomes [15, 37–40]. A similar pattern was observed for γδ T cells, the C14-γδ-TIGIT was the largest such subtype available in patients with DLBCL, consistent with findings from our clinical cohort study. This might be attributed to the following aspects, the interaction of TIGIT with its ligands, primarily CD155, suppresses T cell activation and function, enhancing the immunosuppressive capacities of regulatory T cells (Tregs) and inducing exhaustion in cytotoxic T cells [41]. Besides, in accordance with our previous studies, γδ T cells in patients with AML appear out of balance on distribution of *TIGIT* and *CD226*, accompanying increased TIGIT^+^ CD226^−^ γδ T cells [38]. After chemotherapy, TIGIT^+^ CD226^−^ γδ T cells decreased in AML patients who achieved complete responses (CR) [38], thereby emerging as a potential prognostic or risk factor [38]. These results are consistent with the findings of this study in DLBCL patients, suggesting that TIGIT^+^ γδ T cell subtypes are characterized by high expression of *TIGIT* and low expression of *CD226*. In contrast to *TIGIT*, *CD226* plays a pivotal role in the initiation and stimulation of T cells, thereby augmenting their cytotoxicity, particularly in the presence of PVR-expressing within tumor microenvironment. However, TIGIT competes with CD226 for ligand binding, potentially inhibiting the functionality of T cells [42].

Nevertheless, we also observed discrepancies upon further analysis of C14-γδ-TIGIT. We speculated that in the DLBCL microenvironment, circulating TIGIT^+^ γδ T cells exhibit a multifaced state; they are cytotoxic (on par with C13-γδ-GNLY) yet al.so prone to exhaustion. According to our analysis of cell trajectories, we assumed that γδ T cells gradually differentiate towards terminal states along the pseudo-time trajectories. In relative terms, C13-γδ-GNLY, γδ-TIGIT.1 and γδ-TIGIT.2 are terminally differentiated and gradually incapable of cell stemness as a result of the loss of TCF-1 expression. Relatively, however, γδ-TIGIT.1 exhibited high expression of *CD69*, *IFNG*, *GNLY* and stemness related genes (*TCF7*, *LEF1*, *LTB*). This finding aligns with previous research, which indicated that *CXCR4* is highly expressed on T cells with differentiation potential, but its expression is subsequently downregulated upon T-cell activation [43–46]. The presence of native *CXCR4* has also been demonstrated to enhance T cell immunotherapy by stabilizing immune synapses, suggesting a potential good prognostic marker on T cells [47, 48].

Aside from *CXCR4*^*hi*^ (γδ-TIGIT.1) subtype, the other *TIGIT*^*+*^ subtype was *CX3CR1*^hi^ (γδ-TIGIT.2). Compared with γδ-TIGIT.1 (*CXCR4*^hi^), γδ-TIGIT.2 (*CX3CR1*^hi^) both highly expressed cytotoxic, activation genes (*GNLY*, *GZMB*, *PRF1*, *KLRG1*, *KLRD1*) and immune inhibition genes (*TIGIT*, *TOX*, *LAG3*, etc.). As corroborated by Carmen Gerlach, high expression of *CX3CR1* can be identified as a marker for effector memory T cells (Tem) [49]. Moreover, *CX3CR1*^+^ T cells demonstrate strong cytotoxic activity, whereas *CX3CR1–* T cells are predominantly non-cytotoxic and have higher proliferative potential [50]. The observation from Gerlach was applied to explain our result, yielding another reason that may have resulted, almost all *KLRG1*^*+*^ T cells were *CX3CR1*^*hi*^ [49]. As postulated to be the marker of senescence, *KLRG1* is highly expressed on γδ-TIGIT.2, demonstrating their cell-division ability is progressively lost, resulting in proliferative arrest and apoptosis [51].

Of great interest, we also observed a dichotomous role for TIGIT^+^ γδ T cells in terms of the cytotoxic mediator (*PRF1*, *GZMB*, and *GNLY*) and IFN-γ expression. For instance, higher expression of *TIGIT* on γδ T cells (particularly γδ_TIGIT.2) indicates an exhaustion population. However, such γδ T cells exhibit enhanced cytotoxic function by upregulating *PRF1*, *GZMB*, and *GNLY* and impaired function with diminished *IFNG*. Such dichotomous role has previously been reported in NK cells of HIV patients [52]. Nevertheless, our findings provide a previously unidentified, as we can tell, role for TIGIT in DLBCL pathogenesis, potentially elucidating how the cytotoxicity of γδ T cell subtypes can be improved in DLBCL patients.

Thus, another striking discovery in TIGIT^+^ γδ T cells is that the γδ_TIGIT.1 subtype (*TCF-1*^hi^, *GZMB*^lo^) exhibits similarities in gene expression with the Tpex (precursor exhausted T cell) subtype, whereas the γδ_TIGIT.2 subtype (*TCF-1*^lo^, *GZMB*^hi^) appears akin in gene expression to the Ttex (terminal exhausted T cell) subtype, reminiscent of the Ttex and Tpex subpopulations observed in CD8^+^ T cells [53–55]. This finding implies that we may have identified potential markers for the first time that could denote the Tpex and Ttex subtypes within γδ T cells in DLBCL patients [53, 54]. Moreover, this observation substantiates that with the sequential exposure to tumor antigens, γδ T cells progressively lose *TCF-1* expression. This occurs concurrently with the upregulation of a variety of inhibitory receptors, leading to an incapacity for functional recuperation in γδ T cells [27, 56]. Moreover, in chronic infections and cancer, the *TCF-1 (TCF-7)* in supporting the stemness and self-renewal of T cells is of utmost importance [57]. *TCF-1* serves as a key factor in preventing the exhaustion of T cells during prolonged and intense immune challenges. Its function lies in the preservation of functionality and reactivity of T cells, exerting a substantial influence on the outcomes of ongoing struggles against chronic infections and cancer, thereby providing a foundation for the sustained immune defense mechanisms of the body [58]. Consequently, despite remaining viable in vivo, the diminished expression of *TCF-1* in γδ T cells will progressively render non-functional.

Of significant clinical importance, our study revealed that the active transcriptional factors in the γδ_TIGIT.2 subtype are involved in regulating exhaustion and terminal differentiation [59–61]. Specifically, we identified *TBX21*, *STAT1*, *EMOS*, *SREBF2*, *POLR2A* and *ZBTB7A* as the predominant transcription factors associated with γδ_TIGIT.2, indicating their potential role in governing the exhaustion differentiation of γδ T cells [62]. Additionally, the presence of the γδ-TIGIT.2 subtype is characterized by high expression of *CX3CR1* and low expression of *TCF-1*. Previous studies reported that *TCF-1*^−^*CX3CR1*^+^ exhausted T cells, exhibit the utmost effector function and play a crucial role in exerting a certain degree of control during chronic viral infections [63, 64]. Since *TCF-1*^+^ Tpex cells align with the characteristics of our γδ_TIGIT.1 subtype, it can be inferred that our γδ_TIGIT.2 subtype corresponds to the *TCF-1*^*−*^*CX3CR1*^*+*^ exhausted subtype. Thus, it is possible to speculate that *CX3CR1* and *ITGB1* may potentially participate in regulating the differentiation from the γδ_TIGIT.1 subtype to the γδ_TIGIT.2 subtype through these transcriptional factors with high transcriptional activity in the γδ_TIGIT.2 subtype. A further investigation is ongoing to elucidate the mechanism of those chemokines, transcriptional factors and immune-checkpoints. Additionally, the use of complementary single-cell techniques will be necessary to correlate single-cell phenotypes with the spatial organization of cells within DLBCL. It is crucial to integrate multiple different platforms to fully comprehend the tumor microenvironment (TME) heterogeneity in DLBCL and its clinical significance.

Despite belonging to the same ICRs family as PD-1, TIGIT has unique functions, especially in different diseases where they regulate various aspects of immunity [65]. Our findings are crucial in identifying pivotal transcription factors and genes that may regulate the differentiation process of γδ_TIGIT.1 subtype to γδ_TIGIT.2 subtype, thereby enhancing the comprehension of the intricacies involved in γδ T cell differentiation dynamics.

While this study provides valuable insights into the role of TIGIT in γδ T cells, several important limitations need to be addressed in future research. One key limitation is the lack of a direct functional comparison between γδ T cells from DLBCL patients and healthy donors. This comparison is crucial for understanding the potential alterations in γδ T cell functionality in the context of DLBCL and the effects of TIGIT blockade on these cells. Unfortunately, due to resource constraints and the complexity of obtaining patient samples, this aspect could not be fully explored in the current study. Future work should focus on detailed functional assays comparing γδ T cells from both DLBCL patients and healthy donors, including the evaluation of cytotoxicity, cytokine production, and surface marker expression.

## Conclusions

In our study, we have identified 6 distinct subpopulations of γδ T cells, among which two exhibited features of exhaustion, characterized by the expression of immunosuppressive factors such as *TIGIT*. These particular subtypes have been categorized as Ttex and Tpex clusters, distinguished by their differentiation trajectories and associated cytokine expression profiles. Notably, these γδ T cell subpopulations might have disparate effects on the prognosis of patients with DLBCL, exerting divergent effects on clinical outcomes. In summary, our findings provide novel insights into the genotypic and characteristics of γδ T cells and propose a potential approach for immunotherapeutic intervention and prognostic assessment in the context of DLBCL.

## Electronic Supplementary Material

Below is the link to the electronic supplementary material.

Supplementary Material 1

## Data Availability

The data involved in this study are available from the corresponding author upon reasonable request.

## Abbreviations

AML: Acute Myeloid Leukemia
CR: Complete Response
CTLA4: Cytotoxic T-Lymphocyte Associated Protein 4
CX3CR1: C-X3-Chemokine Receptor 1
CXCR4: C-X-C Chemokine Receptor Type 4
DLBCL: Diffuse Large B-cell Lymphoma
FACS: Fluorescence-Activated Cell Sorting
GZMK: Granzyme K
GO: Gene Ontology
GSVA: Gene Set Variation Analysis
HI: Healthy Individuals
ICR: Immune Checkpoint Receptors
KEGG: Kyoto Encyclopedia of Genes and Genomes
IFN-γ: Interferon gamma
LAG3: Lymphocyte-Activation Gene 3
MACS: Magnetic-Activated Cell Sorting
ND: Newly Diagnosed
NK cells: Natural Killer Cells
PCA: Principal Component Analysis
PDCD1: Programmed Cell Death Protein 1
PFS: Progression-Free Survival
R: R Programming Language
R/R: Relapsed/Refractory
RSS: Regulon Specificity Score
SCENIC: Single-cell regulatory network inference and clustering
scRNA-seq: Single-cell RNA Sequencing
Tem: Effector Memory T Cells
TCF-1: T Cell Factor 1
TME: Tumor Microenvironment
Tpex: Precursor Exhausted T Cells
Ttex: Terminal Exhausted T Cells
Tregs: Regulatory T Cells
TNF: Tumor Necrosis Factor
UMAP: Uniform Manifold Approximation and Projection
UMI: Unique Molecular Identifier
γδT cells: Gamma Delta T Cells
