# Supplementary material for: Heterogeneous characteristics of γδ T cells in peripheral blood of diffuse large B-cell lymphoma
Source: Biomark Res. 2025 Jun 7;13:82. doi: 10.1186/s40364-025-00795-x (PMC12145656; doi:10.1186/s40364-025-00795-x)
Supplement: Supplementary file 1 — Supplementary Material 1 [file 40364_2025_795_MOESM1_ESM.docx]

**Supplementary Figure 1**


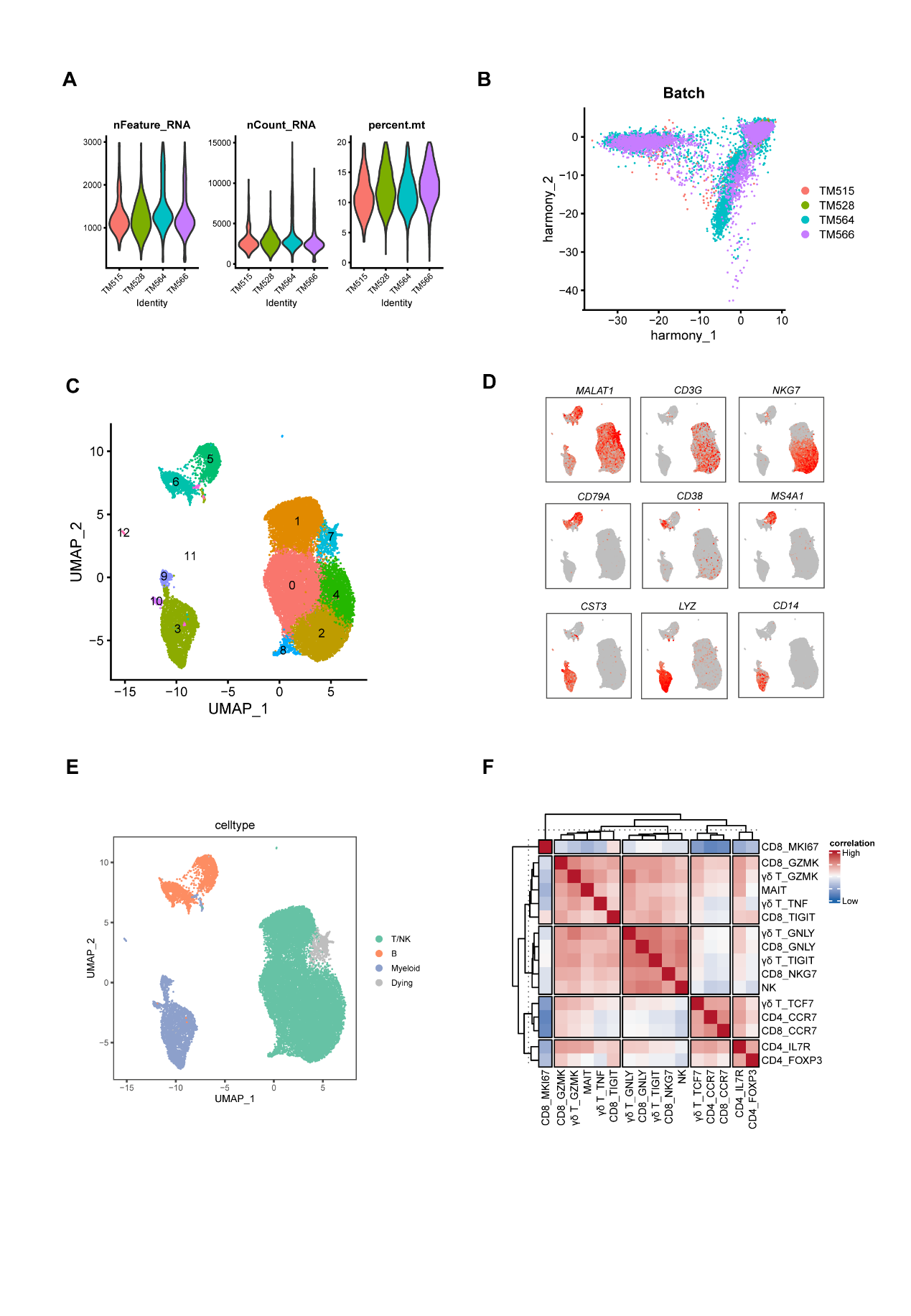
 **Supplementary Figure 1. Quality control of scRNA data.** (A) Quality control was performed to filter the DLBCL cells with poor quality. (B) Harmony and PCA visualization of T cells from combined DLBCL and healthy individuals. Different samples in each plot are depicted with distinct colors. (C) UMAP visualization of PBMC single cell clusters from pan-cancer. Different cell clusters are depicted with distinct colors. (D) Projection of selected marker genes expression level on T cells from DLBCL patients. (E) UMAP visualization of PBMC single cell clusters from DLBCL patients. Different clusters are depicted with distinct colors. (F) Characterization of the T cell clusters using independent reference gene signatures of ‘MonacoImmuneData’ dataset. Heatmap shows cross-labelling of T cell clusters of DLBCL patients deﬁned in the present study (column) versus reference gene signatures (rows) derived from the analyses in ‘MonacoImmuneData’ dataset, with color indicating log-transformed frequency.

**Supplementary Figure 2**


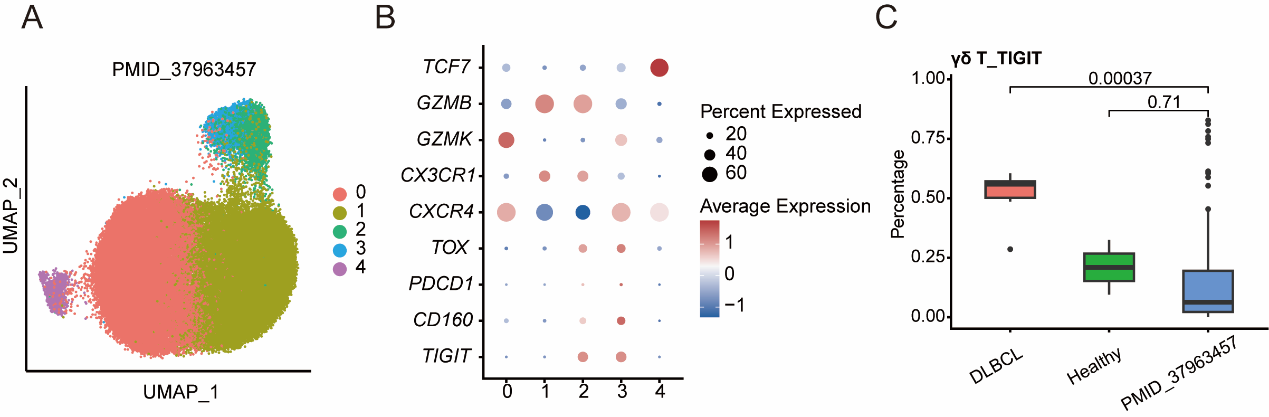


**Supplementary Figure 2.** Re-analysis of scRNA-seq data for peripheral γδ T cells from published literature. (A) UMAP visualization of γδ T cells from the Terekhova M et al. dataset. (B) Dot plot showing the expression levels of marker genes across the clusters. (C) Proportion of the TIGIT^+^ γδ T subset among γδ T cells in our study compared to the published dataset.

**Supplementary Figure 3**

**
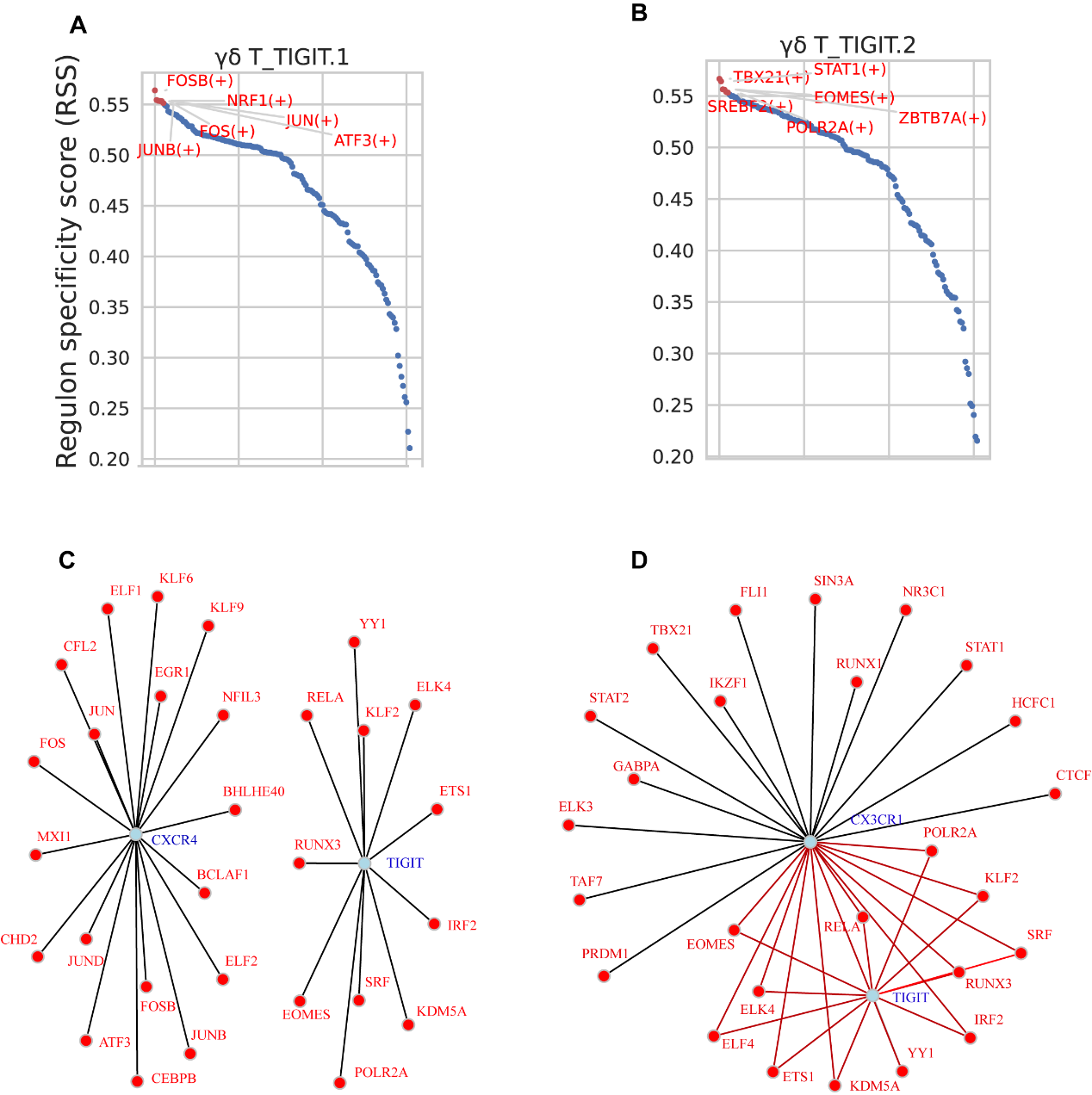
**

**Supplementary Figure 3. Cell-Type-Specific Transcription Factor Regulon Activities and Co-Expression Networks in γδ_TIGIT Subtypes** (A) The regulon specificity score (RSS) reflects top cell-type-specific TF regulon activities of γδ­_TIGIT.1 at single-cell resolution. (B) The regulon specificity score (RSS) reflects top cell-type-specific TF regulon activities of γδ­_TIGIT.2 at single-cell resolution. (C) The co-expression network of TFs with high regulon activation in γδ­_TIGIT.1 and differential genes highly expressed in γδ­_TIGIT.1. (D) The co-expression network of TFs with high regulon activation in γδ­_TIGIT.2 and differential genes highly expressed in γδ­_TIGIT.2.

**Supplementary Figure 4**


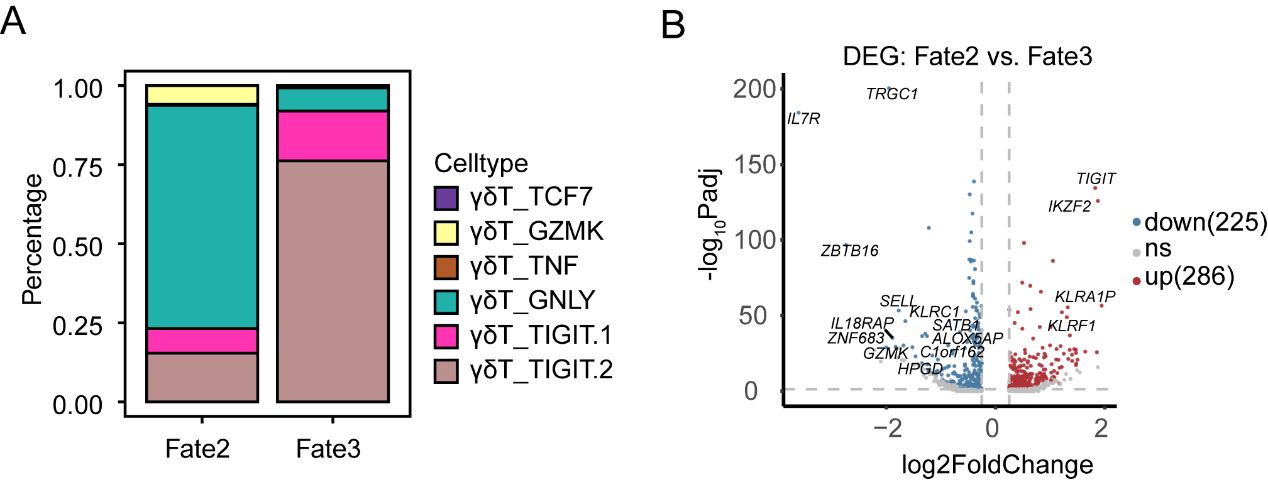


**Supplementary Figure 4. Comparison between cell Fate2 and Fate3 of pseudotime analysis.** (A) Proportion of γδ T cell subsets on Fate2 and Fate3. (B) Different expressed genes between Fate2 and Fate3.

**Supplementary Figure 5**


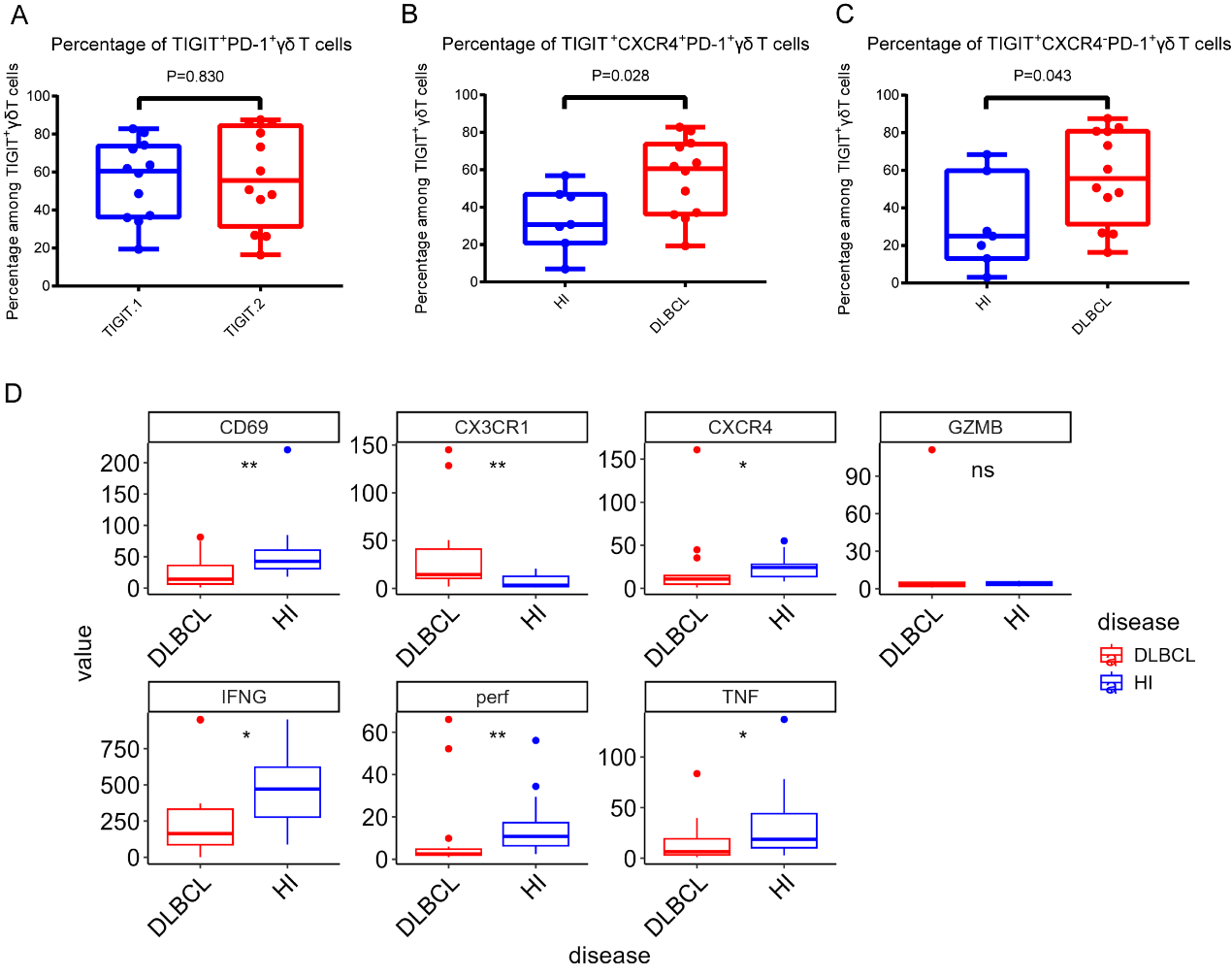


**Supplementary Figure 5. The expression of key genes validated by flow cytometry and qRT-PCR.** (A) Flow cytometry showing the PD1^+^ γδ T cells proportion in γδ­_TIGIT.1 and γδ­_TIGIT.2. The comparison of PD1^+^ (B) γδ­_TIGIT.1 and (C) γδ­_TIGIT.2 between HIs and DLBCL patients. (D) The expression of key genes validated by flow cytometry and qRT-PCR.
